# VARIATIONS IN COGNITIVE STATUS IN OLDER ADULTS WITH MEMORY DIFFICULTIES: THE ROLE OF PERSONALITY AND RESILIENCE

**DOI:** 10.1093/geroni/igac059.2093

**Published:** 2022-12-20

**Authors:** Elizabeth Barbour, Kodi van Antwerp, Majse Lind, Amanda Morgan, Susan Bluck

**Affiliations:** University of Florida, Royal Oak, Michigan, United States; University of Florida, Gainesville, Florida, United States; University of Florida, Gainesville, Florida, United States; University of Florida, Gainesville, Florida, United States; University of Florida, Gainesville, Florida, United States

## Abstract

By approximately 70-years-old, two out of three Americans experience some cognitive impairment (Hale et al., 2020). Cognitive abilities that often decline with age include working and short-term memory (Cohen, 2019), both important for encoding and retaining information (Alloway & Copello, 2013). Depending on severity, affected individuals may face difficulties performing daily tasks. Beyond biological mechanisms, Self-Life Acceptance (Resilience; Wagnild & Young, 1993) and personality (i.e., Neuroticism, Openness; BFI-2-XS; John & Soto, 2017) may relate to variations in cognitive status. We collected measures of Self-Life Acceptance, Neuroticism, and Openness to investigate their relations to older adults’ cognitive status (i.e., working and short-term memory; TICS; Brandt et al., 1988). The sample was comprised of older adults clearly experiencing memory difficulties (N = 49, Mage = 76.12). In a hierarchical regression, the interaction between Self-Life Acceptance and Neuroticism predicted higher cognitive status. Deconstructing this effect, for older people with low-to-moderate Neuroticism, having worse cognitive status was related to greater feelings of Self-Life Acceptance. These individuals show resilience; when cognitive status is worse, acceptance of oneself and life appears to ‘kick in’ allowing individuals to maintain well-being in the face of memory difficulties. Self-Life Acceptance, however, is not present for those high in Neuroticism. In a second regression, less Self-Life Acceptance and higher Openness were also related to better cognitive status. Our findings show psychosocial factors can predict variations in cognitive status. This work provides a window into how older individuals with different personality traits and varying capacity for resilience cope with memory loss.

